# Savory and Peppermint Essential Oils-Loaded Emulsions and Nanoemulsions Effects on *Enterococcus faecium* Isolated from Vacuum-Packed Cured Sausage

**DOI:** 10.3390/foods13020341

**Published:** 2024-01-22

**Authors:** Hadi Hashemi, Ehsan Shad, Fatemeh Ghiasi, Mohammad Hadi Eskandari

**Affiliations:** Department of Food Science and Technology, School of Agriculture, Shiraz University, Shiraz 71441-13131, Iran; hadihashemigahruie@shirazu.ac.ir (H.H.); ehsanshad@shirazu.ac.ir (E.S.); fatemehghiasi@shirazu.ac.ir (F.G.)

**Keywords:** antibacterial activity, nanoemulsion, *Enterococcus faecium*, savory and peppermint essential oil, sliced vacuum-packed cured emulsion-type sausage

## Abstract

In this work, *Enterococcus faecium,* the specific spoilage organism responsible for bloating spoilage of sliced vacuum-packed cured emulsion-type sausage, was isolated and identified through molecular and biochemical techniques, and then the antibacterial activities of savory-loaded nanoemulsion (SNE), savory-loaded emulsion (SE), peppermint-loaded nanoemulsion (PNE), and peppermint-loaded emulsion (PE) were investigated against spoilage microorganisms. Nanoemulsions with average particle sizes in the range of 109.27 to 118.55 nm were developed by sonication and remained more stable than emulsion samples for 2 weeks. Regardless of emulsion type, the highest antimicrobial activity was detected for savory-loaded samples. Moreover, the significant enhancements in the antimicrobial activity of SNE compared to SE were confirmed by increasing the inhibition zone diameter (17.6%) and decreasing MIC (50%) and MBC (50%) due to the higher specific surface area of smaller droplets. The TEM and SEM micrographs confirmed the inhibitory effects of SNE due to the significant changes in the cell wall integrity of *Enterococcus faecium*.

## 1. Introduction

Sliced vacuum-packed cured emulsion-type sausages are considered attractive meat products among consumers and can be stored for up to 30 days under standard refrigerated conditions (8 ± 2 °C). The highly perishable nature of these products is mainly attributed to the low salt concentration (~1.5%), neutral pH (~6.0), and high water activity in the range of 0.95 to 0.97 [1,2]. Lactic acid bacteria (LAB) are considered the main spoilage organisms in cured sausages, due to the presence of nitrite and nitrate as the curing agents. Nitrite inhibits the growth of a wide variety of harmful and spoilage bacteria such as *Clostridium botulinum*, *Bacillus cereus*, *Staphylococcus aureus*, and *Clostridium perfringens* [3]. Homofermentative LAB are strongly resistant to nitrite up to 200 ppm in processed meat products [4], resulting in spoilage during storage. This fact can be attributed to the reduction of nitrite and nitrate via nitrate and nitrite reductase activity, which is considered as the detoxification mechanism [5]. However, this resistance is strain-dependent. Changes in color, odor, texture, and gas production are the main signs of spoilage in cured emulsion-type sausages [2].

Essential oils (EOs), as natural flavoring additives in food formulations, are secondary metabolites of plants [6]. The antibacterial potential of EOs against different pathogens and spoilage bacteria has been well documented in previous research [7,8]. However, the required concentration of EOs to present efficient antimicrobial activity generally exceeds the sensorial acceptance levels. Moreover, other limitations, including poor aqueous solubility, extreme volatility, low stability, and possible interactions with food ingredients, make them difficult to incorporate directly into food formulations [9,10]. Therefore, encapsulation of EOs in emulsion systems can enhance their biological properties in food systems. Compared to conventional emulsions, nanoemulsions with uniform and small droplet sizes, typically less than 200 nm, represent several advantages in terms of better physicochemical stability, enhancement of the biological activities, and bioavailability of bioactive components through improving the specific surface area and hence the reduction of the effective dose of active ingredients, which make them ideal delivery carriers [8,11,12]. The effect of encapsulating EOs in nanoemulsions for enhancement of their antibacterial efficacy in comparison with conventional emulsions has been previously reported for carvacrol [13], *Zataria multiflora* [8], cinnamaldehyde [14], lime [15], sage, lemongrass, and thyme EOs [16].

To our knowledge, there are no published works regarding the antibacterial action of encapsulated savory and peppermint EOs against spoilage bacteria in cured sausages. To this end, biochemical and molecular identification techniques were applied to detect the spoilage LAB bacteria in collected samples. Moreover, the antimicrobial activities of encapsulated EOs in nanoemulsions in comparison with conventional emulsions were explored to purpose an efficient delivery system for food formulations.

## 2. Materials and Methods

### 2.1. Samples Collection

Fifteen different vacuum-packed cured emulsion-type sausages were purchased from a local market in Shiraz, Iran. Samples were kept in the laboratory at 8 °C for 4 weeks and were cultivated via the pour plate technique. In general, 49 colonies were isolated from samples using MRS medium during storage and were then tested through molecular and biochemical techniques, as described in the following sections:

### 2.2. Isolation of LAB Strains

#### 2.2.1. Biochemical Characterization of Isolated Strains

The Gram reaction and spore detection were investigated by staining the isolated bacteria. Moreover, they were evaluated in terms of catalase and oxidase activity, the production of gas from glucose and pentose, and the fermentation of carbohydrates. The phenotypic properties of LABs were also studied using a method modified by Khorsandi et al. [1]. Finally, three different colonies of the most abundant bacteria were chosen for further identification.

#### 2.2.2. DNA Extraction of Isolated Strains

DNA extraction was performed by centrifugation of 1 mL of the isolated bacteria at 13,000× *g* at 4 °C for 15 min. The obtained pellet was dissolved in 0.1 mL of buffer containing Tris HCl (10 mM), acetic acid (20 mM), and EDTA (1 mM) at pH 8.0. According to the procedure for DNA purification from gram-positive bacteria presented by the manufacturer’s instructions, DNA was extracted using a kit (CinnaGen, Tehran, Iran). The extracted DNA was then washed with buffer and kept in a freezer set at −18 °C.

#### 2.2.3. Molecular Identification

Molecular identification was determined using the PCR amplification method of isolated 16s rRNA genes reported by Khorsandi et al. [2]. The sequencing of the samples was performed by Sangon Co., (Shanghai, China) and then analyzed with ChromasPro software.

### 2.3. Essential Oil Extraction

The savory and peppermint plants (10 kg) were collected in summer (Fars province, Iran). The extraction of essential oils from aerial parts was performed using a hydrodistillation process in a Clevenger for 180 min, based on the modified technique of Gahruie et al. [8]. The extracted EOs were dehydrated using anhydrous Na_2_SO_4_ and kept in the dark at −20 °C before further evaluation.

#### GC-FID Analysis of EOs

Gas chromatography (GC) analysis was performed using an Agilent gas chromatograph series 7890A (BEIFEN, Beijing, China) equipped with a flame ionization detector (FID) [8]. The analysis of EOs was performed using a fused silica capillary HP-5 column. The detector and injector temperatures were set at 280 °C and 250 °C, respectively. The flow rate of nitrogen as a carrier gas was 1 mL/min. The temperature of the column was increased from 60 to 210 °C at a rate of 4 °C/min, increased to 240 °C at a rate of 20 °C/min, and then maintained isothermally for 8.5 min. The split ratio was 1:50.

### 2.4. Emulsion and Nanoemulsion Preparation

Emulsification was performed according to the method of Hashemi et al. [8], with some modifications. Briefly, an aqueous dispersion of Tween 80 (2.5% (*w*/*w*)) in double-distilled water was prepared, and then savory and peppermint EOs (6% (*w*/*w*)) were added individually into each dispersion and homogenized for 30 min at 700 rpm on a magnetic stirrer. To form a coarse peppermint-loaded emulsion (PE) and savory-loaded emulsion (SE), each sample was homogenized at 15,000 rpm for 4 min using a T18 IKA homogenizer (Germany). For the preparation of nanoemulsions, the prepared coarse emulsions were sonicated using a 20 kHz ultrasonic homogenizer (HD3200, Bandelin, Germany) performed at 150 W for 10 min. The resulting nanoemulsions (peppermint-loaded nanoemulsion (PNE) and savory-loaded nanoemulsion (SNE)) were kept in glass flasks at 4 °C under dark conditions.

#### 2.4.1. Droplet Size and Zeta Potential

The volume-weighted mean droplet size and distribution width (Span) were studied using dynamic light scattering (DLS) (SZ100, Horiba, Kyoto, Japan) at 20 °C.

The electrical surface charge of the samples was investigated by DLS (SZ100, Horiba, Japan) at room temperature according to the Smoluchowski equation (Equation (1)).

(1) $$\mu_{e}=\frac{\varepsilon \;\zeta \;}{\eta \;}$$

where μ_e_ is the electrophoretic mobility (m^2^ s^−1^ V^−1^), ε is the permittivity (J V^−2^ m^−1^), ζ is the zeta potential (V), and η is the viscosity (g m^−1^ s^−1^).

#### 2.4.2. Storage Stability

The storage stability of the samples was evaluated by measuring the changes in droplet size and zeta potential during storage at 4 °C for 14 days.

#### 2.4.3. Gravitational Stability

Gravitational stability was investigated by monitoring the amount of water separation from the samples using centrifugation at 5000× *g* and 20 °C for 35 min.

#### 2.4.4. Antimicrobial Activity

##### Agar Diffusion Method

The antimicrobial activity of the samples was investigated by the agar diffusion method, as described by Ghiasi et al. [11]. Briefly, 0.1 mL of bacterial suspension (1.5 × 10^7^ CFU/mL) was cultured on Mueller–Hinton agar plates. The wells were created on the plate surface by a punch and then filled with 0.1 mL of the sample. After incubation at 37 °C for 24 h, digital pictures were taken from all plates, and the inhibition zone (DIZ; mm) in the pictures was measured using Photoshop CS 6.

##### MIC and MBC

The MIC and MBC were evaluated according to the method of Shahbazi et al. [17]. Firstly, a single colony was inoculated into MRS broth subculture and then incubated overnight at 37 °C (160 rpm). The bacterial concentration was set to 1.5 × 10^7^ CFU/mL and applied as inoculum. Serial dilutions in a microplate were conducted in a concentration ranging from 0.47 to 120 µL/mL of samples and then kept at 37 °C overnight. The formation of a pellet at the well bottom and the increase in turbidity were indications of bacterial growth. To determine MBC, 0.1 mL of each sample with no visible signs of turbidity was cultured on MRS agar, and then plates were kept for 48 h at 37 °C.

##### Antibacterial Dynamics

The antibacterial dynamics were evaluated using the microdilution technique according to Section 2.4.2. Briefly, the *Enterococcus faecium* was incubated at 37 °C with continuous shaking at 150 rpm, followed by the determination of absorbance at 600 nm every 60 min for 24 h (25 times) using a CYTATION 3 microplate reader (USA). Antimicrobial activity was reported as the area under the curve (AUC) using Equation (2).

(2) $$\mathrm{AUC}=(\overset{n-1}{\underset{i=1}{\sum }}\frac{\left(m_{\left(i+1\right)}+m_{i}\right)}{2})-(m_{i}\overset{n-1}{\underset{i=1}{\sum }}t_{i})$$

where m_i_ is the absorbance at t_i_ and m_(i+1)_ is the absorbance at t_(i+1)_.

#### 2.4.5. Transmission Electron Microscopic (TEM)

The changes in the structure of *Enterococcus faecium* after treatment with EOs-loaded SE and SNE samples were determined using a TEM. The untreated sample was also prepared as the control. The isolated bacteria were cultured at 37 °C in MRS broth for 18 h and then each sample was mixed into the bacterial suspension. All samples were incubated at 37 °C for 6 h and then centrifuged to separate the cells. Then, they were washed 3 times with PBS (0.1 M, pH 7.2), fixed with glutaraldehyde (2.5% *v*/*v* in 0.1 M PBS) for 24 h at 4 °C, and post-fixed with an osmic acid solution (1% *w*/*v* in 0.1 M PBS) for 120 min at 25 °C. For the dehydration of the specimens, a series of ethanol solutions (50, 60, 70, 80, 90, and 100%) were used [18]. After staining the dried cells, they were investigated by a TEM (LEO-906E, Carl Zeiss, Jena, Germany) operated at 80 kV.

#### 2.4.6. Scanning Electron Microscope

The morphological changes in cell walls after treatment with EO-loaded emulsions and nanoemulsions were determined using a scanning electron microscope (SEM). The bacterium in the MRS was incubated at 37 °C overnight. The bacterial suspension was divided into 2 parts. One part was left untreated as a control, and SE and SNE samples were added to the remaining part and then incubated at 37 °C for 6 h. Afterward, the cells within each tube were separated by centrifugation. The obtained cells were fixed with 2.5% *v*/*v* glutaraldehyde in 0.1 M PBS and stored at 4 °C overnight. Dehydration of bacterial cells was conducted using a series of ethanol solutions (30, 40, 50, 60, 70, 80, 90, and 100%) for 20 min. They were then freeze-dried (Dena vacuum Dryer-FD-5003-BT, Dena Vacuum, Iran) (−35 °C and 0.001 mbar) for 4 h Chen et al. [14] and observed using a scanning electron microscope (TESCAN-Vega 3, Brno, Czech) after coating with a thin layer of gold (Desk Sputter Coater DSR1, Nanostructural Coating Co., Tehran, Iran). Micrographs were taken at an accelerating voltage of 20 kV and magnification of 5000×.

### 2.5. Statistical Analysis

One-way analysis of variance (ANOVA) and Duncan’s multiple tests were applied for data analysis and determination of the significance of differences (*p* < 0.05) between mean values at the 5% probability level. Numerical experiments were performed at least in triplicate, and the SAS 9.1 program (SAS Inc., Cary, NC, USA) was used for the statistical analysis.

## 3. Results and Discussion

### 3.1. Bacterial Identification

According to the Gram reaction, all LAB isolates were divided into three groups according to their phenotypic similarity. These groups contained twenty-one (group one), fifteen (group two), and thirteen (group three) isolated, respectively. Finally, one colony was chosen from each group for further molecular identification. The obtained results indicated that *Enterococcus faecium* and *Lactobacillus dextrinicus* were the main species responsible for bloating spoilage in sliced vacuum-packed cured emulsion-type sausage, which presented a sequence similarity in the range of 97–99% with the GenBank database (Table 1). Moreover, the morphological properties confirmed the results of the molecular analysis. There are similar reports on the identification of spoilage bacteria in meat products using 16S rDNA-DGGE [19,20]. However, the results of Hu et al. [19] regarding the identification of spoilage bacteria in sliced vacuum-packed cooked ham were different from our findings. They isolated 106 colonies from different samples during storage time. The microbiology results showed seventeen unique colonies, while 16S rDNA-DGGE analysis revealed only six different species, and the main isolated bacteria were *Lactobacillus sakei* and *Leuconostoc mesenteroides.* The reason for these different results may be attributed to post-contamination as the main reason for spoilage of sliced vacuum meat products, as well as different local production and processing.

### 3.2. Yield and Chemical Composition of EO

The yields of extraction for savory and peppermint EOs were 1.31% and 0.59%, respectively. The EOs composition, their relative percentages, and retention times (RT) obtained by GC-FID are shown in Table 2. Seventeen compounds were detected in the savory EO. The main constituents were γ-terpinene (82.98%), carvacrol (7.56%), and terpinene-4-ol (2.40%). Memarzadeh et al. [21] also reported that γ-terpinene, carvacrol, and thymol are the main components of savory. The presence of carvacrol and *p*-cymene in savory EO was also reported by Šojić et al. [22]. Twenty-one compounds were identified in the peppermint EO. The main constituents were γ-menthol (30.09%), menthone (23.73%), and 1,8-cineole (17.64%). Similar chemical compositions were also previously observed by Khalvandi et al. [23] and Kang et al. [24] for peppermint EO.

### 3.3. Emulsion Characterization

**Figure 1:**
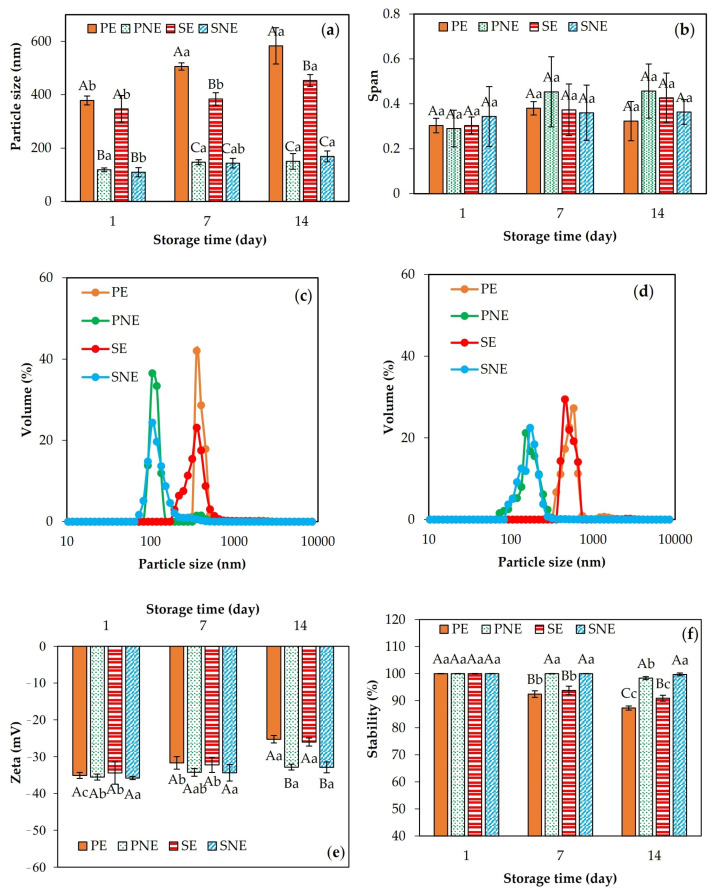
Changes in the particle size (**a**), span (**b**), size distribution ((**c**) (day 1) and (**d**) (day 14)), zeta potential (**e**), and stability (**f**) of peppermint-loaded emulsion (PE), savory-loaded emulsion (SE), peppermint-loaded nanoemulsion (PNE), and savory-loaded nanoemulsion (SNE) as a function of storage time. At the same time of storage, different capital letters indicate significant (*p* < 0.05) differences between different emulsions. For the same type of emulsion, different lowercase letters indicate significant (*p* < 0.05) differences over time.

As the emulsion droplet size can alter the antimicrobial activities of EOs, the effect of sonication on the reduction of droplet size was evaluated. The volume-weighted mean droplet size before and after sonication is presented in Figure 1a. The droplet sizes of fresh emulsions were increased significantly from 348–378 nm to 453–583 nm during storage. Whereas for fresh nanoemulsion samples, sonication led to the formation of droplet sizes in the range of 109–118 nm, which had less increment at the end of storage (149–168 nm). Generally, the droplet size of nanoemulsions formed by sonication can be affected by different operation parameters, including power, amplitude, time, and temperature [12,25]. The major mechanism for emulsification using sonication is the application of a high shear force induced by acoustic cavitation, which leads to significant droplet disruption, facilitating the formation of stable and small droplets [26]. Similar observations were also reported by Gahruie et al. [8] for *Zataria multiflora* essential oil (ZMEO)-loaded nanoemulsion under the effect of sonication time, Jafari et al. [27] for d-limonene-loaded nanoemulsions prepared with microfluidization and sonication, and Hashtjin and Abbasi [28] for orange peel essential oil-loaded nanoemulsions developed in different sonication conditions (time and intensity). According to Figure 1b, the distribution width (span) of fresh samples was in the range of 0.29–0.34, which remained relatively constant during storage. Therefore, sonication had no significant effect on the size of distribution (Figure 1c,d). On the other hand, the relatively high negative charge in all fresh samples (Figure 1e) confirmed the immediate stability after preparation. Since the thermodynamic stability of emulsions is required for most industrial applications, changes in zeta potential were also investigated over 2 weeks (Figure 1e). Irrespective of emulsion type, the zeta potential values for samples remained relatively constant after one week of storage. However, a significant reduction was observed after two weeks for the emulsion samples. While nanoemulsions were completely stable for up to 14 days. Stability analysis also confirmed the results of the zeta potential measurements (Figure 1f). For peppermint- and savory-loaded emulsions, the stability decreased significantly to 87% and 90% after two weeks, respectively. No phase separation was observed for the nanoemulsions at the end of storage, which was proof of the good kinetic stability of the nanoemulsions obtained by sonication (Figure 1f).

### 3.4. Antibacterial Assay

#### 3.4.1. Agar Diffusion Method, Minimum Inhibitory Concentration, and Minimum Bactericidal Concentration

Table 3 presents the results of the agar diffusion test for peppermint- and savory-loaded emulsions and nanoemulsions. Generally, the inhibition zone diameter values below 8 mm, 8–14 mm, 14–20 mm, and above 20 mm were characterized as not sensitive, sensitive, very sensitive, and extremely sensitive bacteria, respectively [29]. The EOs-loaded nanoemulsions presented larger zones of inhibition than the emulsion ones, indicating their greater effectiveness for interactions between active compounds of EOs with biological membranes. This means that the high surface tension of nanoemulsions led to an increase in antimicrobial activity due to better transfer through the cell membrane [8]. On the other hand, both the SNE and SE showed stronger antibacterial activity against *Enterococcus faecium* compared to the peppermint-loaded samples. In general, the antimicrobial activity of EOs was affected by the chemical composition and the functional groups present in bioactive components. High antimicrobial activity of savory EO was previously observed by Abdollahi et al. [30], Atef et al. [31], and Feyzioglu and Tornuk [32]. γ-Terpinene is one of the main antibacterial components of savory [21,30]. The main antibacterial actions of γ-terpinene can be attributed to the inhibition of ATPase activity, membrane disruption and destabilization, fluidization of membrane lipids, leakage of cell ions, and decrease in proton motive force [33,34]. Other antimicrobial components in the savory EO were α-pinene [35], terpinene-4-ol [36], and carvacrol [37], which were in good agreement with previous reports. The antimicrobial activity of peppermint EO was also mainly attributed to 1,8-Cineole [38], *cis*-Sabinene hydrate [39], menthone [40], isomenthone [41], and menthol [42]. Generally, different EOs extracted from different plant materials, including fruits, bark, seeds, pulp, peel, root, and whole plants, contain various antibacterial compounds such as aromatic hydrocarbons, aldehydes, ketones, terpene, terpenoids, esters, alcohols, and acids [43].

The results of MIC and MBC (Table 2) were also in good agreement with the diameter of the inhibition zones. The most effective treatments against *Enterococcus faecium* were savory-loaded samples, and the least effective were peppermint-loaded ones with the highest MIC and MBC values.

#### 3.4.2. Antibacterial Dynamics

**Figure 2:**
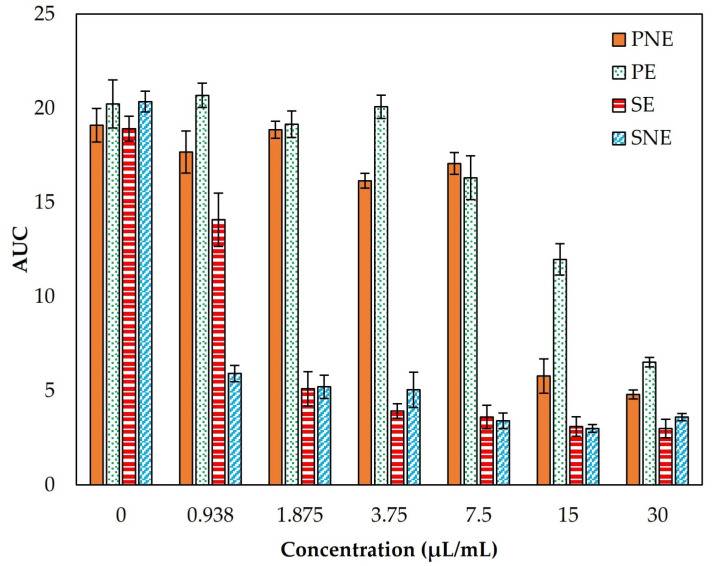
The area under curves (AUC) obtained from antibacterial assay on peppermint-loaded emulsion (PE), savory-loaded emulsion (SE), peppermint-loaded nanoemulsion (PNE), and savory-loaded nanoemulsion (SNE) by microplate reader against *Enterococcus faecium* after 24 h.

The area under the growth curves (AUC) of *Enterococcus faecium* as affected by different sample concentrations (0–30 µL/mL) are shown in Figure 2. The antibacterial properties of all samples progressively increased with increasing concentrations of peppermint and savory EOs. The obtained results supported that savory EO presented a more inhibitory effect on the growth of *Enterococcus faecium* than peppermint, as discussed previously. Moreover, the antibacterial potential of nanoemulsions was significantly stronger than that of emulsions, which could be related to the better physical stability of encapsulated EOs and more homogenous dispersion of EOs droplets in the aqueous phase at the nanoscale [44].

#### 3.4.3. Transmission Electron Microscopy

**Figure 3:**
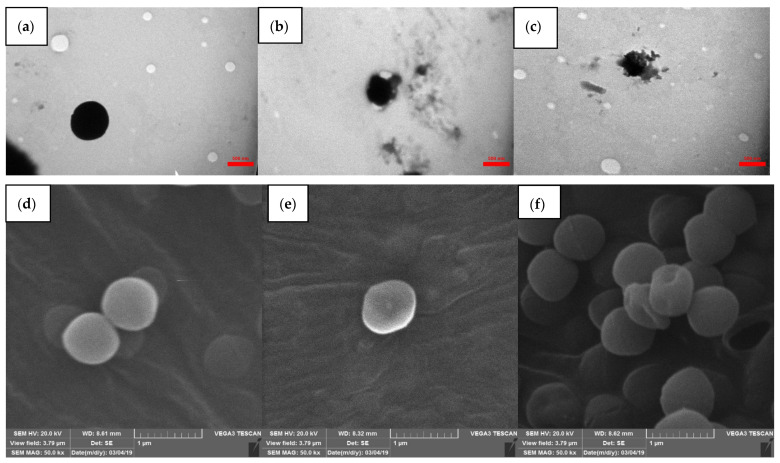
Transmission electron micrograph (**a**–**c**) and scanning electron micrograph (**d**–**f**) of untreated *Enterococcus faecium* (**a**,**d**), treated with savory emulsion (**b**,**e**), and treated with savory nanoemulsion (**c**,**f**).

Since the antimicrobial results showed better performance of savory than the peppermint essential oil, savory-loaded samples were selected for investigation of the cell structure of *Enterococcus faecium* using TEM. The TEM micrograph of untreated cells of *Enterococcus faecium* had a normal cocci-shaped form with complete, slightly corrugated, and well-defined cell walls (Figure 3a). After exposure of *Enterococcus faecium* to savory-loaded emulsion and nanoemulsion, the cell wall seemed swollen and severely injured with changes in morphology, demonstrating a loss of membrane integrity (Figure 3b,c). Additionally, the damaged cells exhibited rougher and unclear membrane boundaries. Ziaee et al. [45] also reported the antimicrobial activity of ZMEO oil against *Lactobacillus curvatus* by the disruption of the membrane in TEM micrographs.

#### 3.4.4. Scanning Electron Microscopy

SEM images can provide good information about morphological changes in *Enterococcus faecium* membranes after treatment with savory-loaded emulsion and nanoemulsion. As can be seen in Figure 3d,f, the untreated bacteria had a smooth cell contour, while the treated *Enterococcus faecium* showed destructive effects and morphological changes with visible indentions. The results of microscopic images confirmed the harmful effects of savory emulsion and nanoemulsion on the cell wall of treated bacteria compared to the control group. Chen et al. [46] also reported a regular, healthy manner with a smooth surface for untreated *E. coli*, while cells were deformed, pitted, and wrinkled with visible membrane degradation after treatment with ginger essential oil. Moreover, Zhang et al. [47] and Chauhan and Kang [48] observed similar effects of cinnamon essential oil on *S. aureus* and *E. coli*. and thymol against *S. typhimurium*, respectively.

## 4. Conclusions

The current study focused on the formation, characterization, and comparison of EOs-loaded conventional emulsions and nanoemulsions against the main bacteria associated with meat product spoilage. First, *Enterococcus faecium* and *Lactobacillus dextrinicus* were identified using 16S rDNA analysis and DNA extraction as the main LAB in vacuum-packed cured sausages. Based on our research, savory-loaded nanoemulsion, which was rich in γ-terpinene, carvacrol, and terpinene-4-ol, and peppermint-loaded nanoemulsion, which was rich in γ-menthol, menthone, and 1,8-cineole, possessed superior antibacterial activity against *Enterococcus faecium* compared to the respective emulsions due to their smaller droplet size (109.27 to 118.55 nm), higher surface area, and hence lower surface tension. The high negative zeta potential of nanoemulsions in the range of −35.57 to −35.80 nm confirmed the good stability of nanoemulsions. Moreover, the highest diameter of the inhibition zone and the lowest minimum inhibitory concentration and minimum bactericidal concentration were found for SNE against *Enterococcus faecium*. Moreover, the TEM and SEM observations also confirmed the stronger effect of savory-loaded nanoemulsion on changing the morphology and structure of *Enterococcus faecium* cells. However, further studies are required to find the main mechanisms against other pathogens to justify the real applications of savory as a natural preservative in water-rich food formulations with high functionality and stability.

## Figures and Tables

**Table 1:** Molecular and morphological analysis of LAB isolated from vacuum-packed sliced cured emulsion-type sausage.

	Molecular Analysis				Morphological Analysis ^a^					
Group No.	Bacteria	ID%	Strain Code	Accession No.	Pentose Fermentation	Gas Production	Catalase	Oxidase	Gram Reaction	Spore
1	*Enterococcus faecium*	97	HHG6801	Mk757986	-	-	-	-	+	-
2	*Lactobacillus Dextrinicus*	98	HHG1396	MK757985	-	+	-	-	+	-
3	*Enterococcus faecium*	99	HHG1368	MK447744	-	-	-	-	+	-

^a^—negative reaction to the tests; positive reaction to the tests.

**Table 2:** The major chemical compounds of savory and peppermint EOs.

Savory EO			Peppermint EO		
	RT (min)	%		RT (min)	%
α-Thujene	6.439	0.3379	1,8-Cineole	3.044	17.64
α-Pinene	8.404	1.065	*γ*-Terpinene	3.607	0.9653
β-Pinene	11.494	0.09548	*cis*-Sabinene hydrate	4.629	2.388
β-Myrcene	12.676	0.2385	Linalol	4.847	0.6009
α-Terpinene	14.999	0.8001	Menthone	6.402	23.73
p-Cymene	16.418	0.1942	Menthofuran	8.769	1.729
γ-Terpinene	20.887	82.98	*neo*-Menthol	10.919	1.093
Terpinene-4-ol	21.767	2.404	Isopulegone	11.377	1.359
Thymol	23.226	0.2229	Isomenthone	12.964	12.04
Carvacrol	27.038	7.558	Isomenthol	13.804	2.74
Carvacryl acetate	28.768	0.07459	Menthol	16.001	30.09
β-Caryophyllene	32.618	0.6798	Terpinen-4-ol	16.611	0.2562
Aromadendrene	37.799	0.5519	Pulegone	17.227	0.3644
α-Humulen	42.783	0.5318	Piperitone	18.031	0.2808
β-Bisabolene	47.584	0.4583	*p*-Menth-1-en-9-ol	19.349	1.25
cis-α-Bisabolene	52.127	0.9014	*β*-Bourbonene	19.925	0.5337
Spathulenol	56.935	0.9019	(*E*)-Caryophyllene	21.575	1.283
			(*E*)-*β*-Farnesene	22.997	0.601
			Germacrene D	25.834	0.2366
			Elixene	28.544	0.1623
			Viridiflorol	31.012	0.6576

**Table 3:** Antimicrobial properties of PNE, PE, SE, and SNE against *Enterococcus faecium*.

Treat	MIC (µL/mL)		MBC (µL/mL)		DIZ (mm)
	Emulsion	EO	Emulsion	EO	
PNE	15.00± 3.06 ^B^	900	30.00± 6.12 ^A^	1800	6.30 ± 0.40 ^C^
PE	30.00 ± 6.12 ^A^	1800	30.00 ± 6.12 ^A^	1800	5.10 ± 0.30 ^D^
SNE	1.88 ± 0.39 ^D^	112.8	3.75 ± 0.77 ^C^	225	12.50± 0.70 ^A^
SE	3.75 ± 0.97 ^C^	225	7.50 ± 1.92 ^B^	450	10.30 ± 0.30 ^B^

Data represent the mean ± standard deviation of three independent replicates; different superscript letters in each column indicate significant differences (*p* < 0.05). Peppermint emulsion (PE), Peppermint nanoemulsion (PNE), savory emulsion (SE), and savory nanoemulsion (SNE).

## Data Availability

Data is contained within the article.
